# Thermal Management of GaN-on-Si High Electron Mobility Transistor by Copper Filled Micro-Trench Structure

**DOI:** 10.1038/s41598-019-56292-3

**Published:** 2019-12-23

**Authors:** Srikant Kumar Mohanty, Yu-Yan Chen, Ping-Hung Yeh, Ray-Hua Horng

**Affiliations:** 1 Department of Photonics, National Chiao Tung University, Hsinchu, 30010 Taiwan, ROC; 2 Department of Physics, Tamkang University, New Taipei City, 25137 Taiwan, ROC; 3 Institute of Electronics, National Chiao Tung University, Hsinchu, 30010 Taiwan, ROC; 4 Center for Emergent Functional Matter Science, National Chiao Tung University, Hsinchu, 30010 Taiwan, ROC

**Keywords:** Engineering, Materials for devices

## Abstract

Self-heating effect is a major limitation in achieving the full performance potential of high power GaN power devices. In this work, we reported a micro-trench structure fabricated on the silicon substrate of an AlGaN/GaN high electron mobility transistor (HEMT) via deep reactive ion etching, which was subsequently filled with high thermal conductive material, copper using the electroplating process. From the current-voltage characteristics, the saturation drain current was improved by approximately 17% with the copper filled micro-trench structure due to efficient heat dissipation. The I_DS_ difference between the pulse and DC bias measurement was about 21% at high bias V_DS_ due to the self-heating effect. In contrast, the difference was reduced to approximately 8% for the devices with the implementation of the proposed structure. Using Micro-Raman thermometry, we showed that temperature near the drain edge of the channel can be lowered by approximately ~22 °C in a HEMT operating at ~10.6 Wmm^−1^ after the implementation of the trench structure. An effective method for the improvement of thermal management to enhance the performance of GaN-on-Silicon HEMTs was demonstrated.

## Introduction

Wide-bandgap semiconductors such as gallium nitride (GaN) and silicon carbide (SiC) are considered as outstanding materials for high frequency, high power and opto-electronic devices^1–3^. High power sub-millimeter wave RF and microwave application including satellite communication and 5 G technology are the vital areas, which fueled the growth of GaN based devices^4^. The GaN based power-switching devices offer greater efficiency, power handling and other performances, which attributes that are vital for meeting the high-power, high-density demands of today’s high-end computers, systems and servers. These capabilities are due to two dimensional electron gas (2DEG) properties, which forms at the wide-bandgap GaN/AlGaN interface without any doping^5^.

AlGaN/GaN heterostructure-based high electron mobility transistors (HEMTs) have been widely studied for high-power and high-frequency applications due to their high electron density, high thermal stability, high drift velocity, and large breakdown electric field^6,7^. However, one of the main limitations of their performance is the self-heating effect, which degrades the drain saturation current, transconductance and causes reliability problem. The self-heating effect, which has been well reported in the literature^8–10^, increases the channel temperature due to the transfer of energy from the electrons to the lattice. The increase in the lattice temperature at an elevated drive voltage is due to dissipation of Joule power^11,12^, which causes a drop in the carrier mobility and electron saturation velocity because of the enhanced phonon scattering. In addition, at very high power levels, there is a rapid increase in the junction temperature, which critically affects the mean-time to failure (MTTF) of GaN transistors^13^. Numerous methods have been proposed to improve heat dissipation by using high thermally conductive substrates such as diamond and silicon carbide. However, there is a quick decrease of thermal conductivity with the rise of temperature due to phonon scattering caused by electrically active impurities (Al, N) in SiC and the presence of N impurity in the diamond^14^. Furthermore, these substrates are not very promising in terms of cost and diameter-scalability. Composite substrates^15^ and flip-chip bonding with epoxy under fill^16^ methods have been implemented to decrease thermal impedance on the whole wafer; however, the problem of hot spots in the device channel region continues to exist. In other studies, several approaches towards suppression of the self-heating effect have been investigated, such as substrate transfer^17,18^ and heat spreading using nanocrystalline diamondon the top surface of the device^19,20^. However, devices using these methods have shown moderate performance.

Copper (Cu) filled trench structures in silicon (Si) wafer methods are being developed^21,22^. This could be an effective solution for heat dissipation in vertical direction. Hsueh *et al*. proposed through-substrate via structure on SOI substrate with Ti/Al back side layer to improve heat dissipation. There is no any Cu being filled in the trench^23^. Even local removing the Si substrate, there still existed the self-heating phenomenon. The same behavior has also been reported in the literature reported by P. Srivastava *et. al*.^24,25^. Moreover, Pavlidis *et al*. studied only the thermal characterization using Raman thermometry and transient thermoreflectance imaging method^26^. In this case, AlN with 15 μm thickness was deposited on backside of AlGaN buffer layer after trench etching. Then copper with 2 µm thickness was deposited. The result shows the thermal performance was inferior as compared with that of device with Si substrate without trench structure. The inferior performance could be resulted from the AlN layer deposition after silicon removal and resulted in the thermal resistive increasing. Hwang *et al*. had only shown the simulation study of junction temperature of GaN HEMT with through via hole at different location and size under the active region of the device^27^. In this study, a micro-trench structure filled with high thermal conductivity material, such as copper (390 W/mK), was fabricated on the Si substrate to provide heat escape path from the hot spot. From the electrical characterization with DC and pulsed measurement, significant improvements in the drain saturation current and transconductance were found with the proposed structure. The distribution of temperature throughout the active region (and particularly in the channel region) was measured using IR thermometry. From the measured experimental data, it was clear that the heat generation was highly localized in the channel on the drain side of the gate. With fabrication of micro-trench structure, the temperatures at these hot spots were reduced significantly, which subsequently improved the performance of the AlGaN/GaN heterojunction transistor. Further, non-contact and non-destructive micro-Raman spectroscopy measurements were performed at different drain-source biases (V_DS_) for monitoring the channel temperature. The rise in the channel temperature due to high bias power causes increased phonon scattering and thus degrades the phonon lifetime^28^. Raman thermometry on GaN-based devices estimates shift in the frequency of a single phonon mode, mostly the E2 (high) mode due to its well-known temperature relationship^29^. From the measurement, it was observed that the shift of the E2 (high) peak was lower in the Cu-deposited trench structure, which demonstrated effective heat removal.

## Results

**Figure 1:**
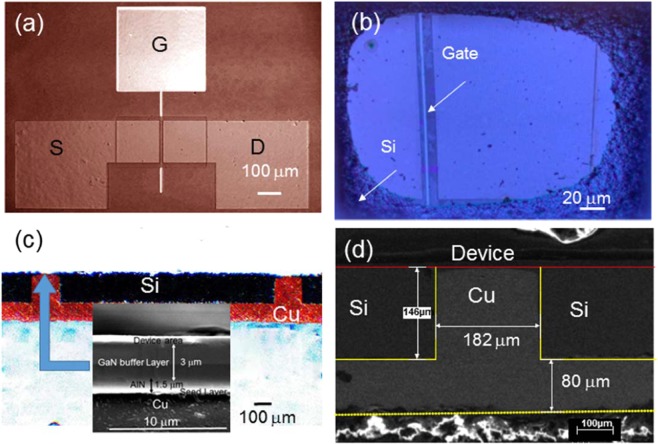
(**a**) OM top view of fabricated GaN HEMT on silicon, (**b**) OM backside view of the HEMT structure after complete removal of the Si, (**c**) OM cross-section view of the copper filled trench structure, and (**d**) SEM cross-sectional view after copper filling in the micro-trench region. The inset of (**c**) is the device structure above the Cu.

Figure 1(a,b) display the optical microscope (OM) top and bottom view of asymmetrical HEMT device after complete removal of silicon. The OM and scanning electron microscope (SEM) images of the cross-section of the micro-trench structure after copper filling are shown in Fig. 1(c,d), respectively. From the figure, it is obvious that Cu not only filled the trench but also covered the whole backside of the Si substrate.

**Figure 2:**
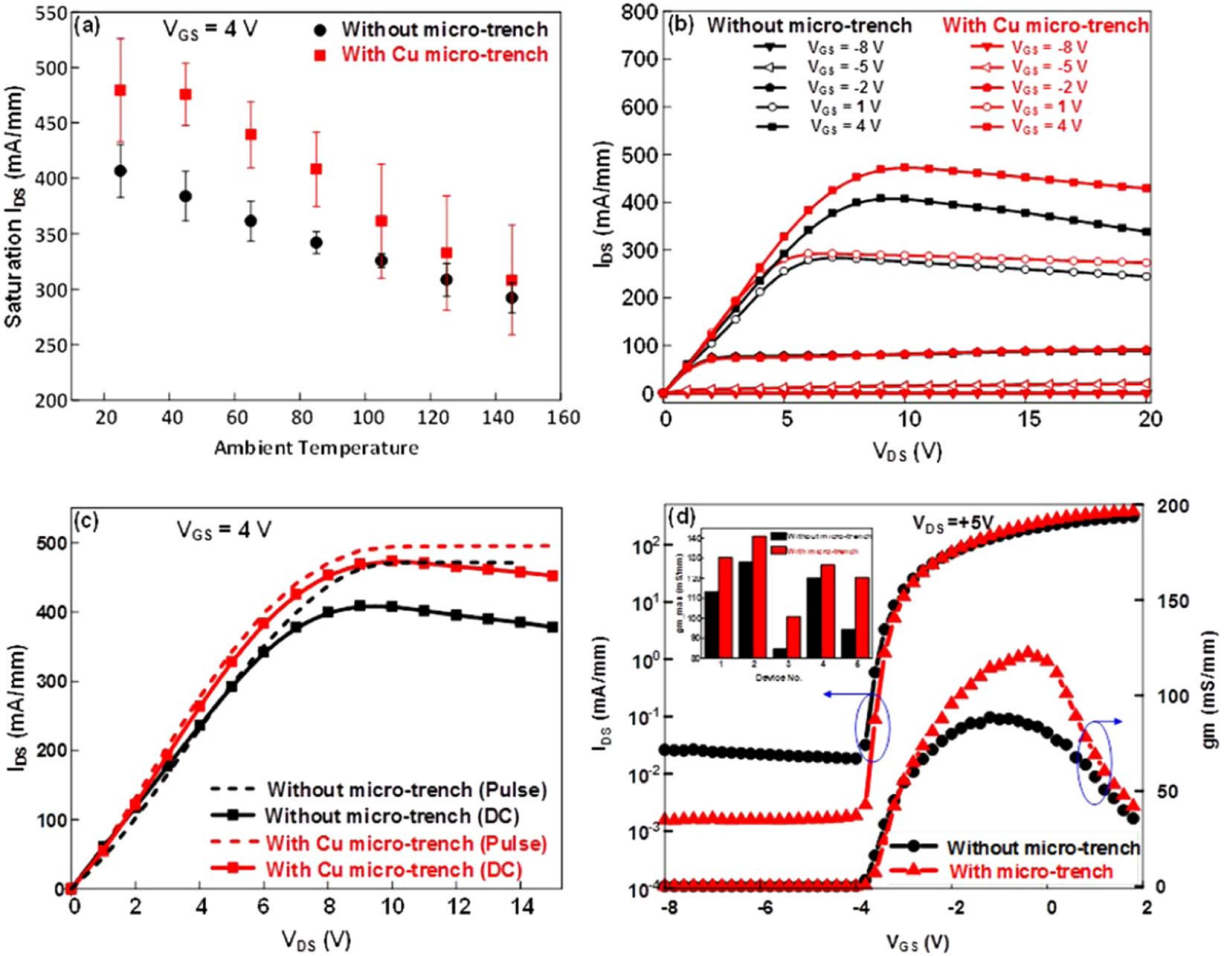
I-V characteristics without and with micro-trench fabrication. (**a)** Saturation current in the HEMT biased at V_DS_ = 20 V as a function of the ambient temperature, **(b)** DC bias current-voltage measurement of the drain current at V_GS_ = −8 V: +3 V step, **(c)** Pulsed measurement of the drain current at V_GS_ = + 4 V with a pulse width of 500 ns and a duty cycle of 0.01%, **(d)** Comparison of the I_DS_-V_GS_ and g_m_-V_GS_ plots without and with a micro-trench. Inset of (**d**) show the g_m_ variation among devices.

Figure 2 presents the current-voltage (I-V) and transfer characteristics of the GaN-on-Si HEMT with micro-trench filled by Cu metal and compared it to the device without micro-trench fabrication (only 150 μm Si thickness). In order to study the temperature effect on the I_DS_, the drain saturation current was examined by changing the ambient temperature through placing the device on a hot chuck. At a gate-source bias of V_GS_ = 4 V, the chuck temperature varied from 25 to 150 °C and the measured the saturation current I_DS_ with the rise in ambient temperature in both devices without and with micro-trench structure, shown in Fig. 2(a). It was found that I_DS_ decreased as the ambient temperature increasing. This reduction of I_DS_ is due to the temperature dependence of mobility and saturation velocity. Even they were both degraded, the I_DS_ for the HEMT with Cu micro-trench was always higher than those of HEMT without micro-trench. It suggests that the performance of HEMT with Cu micro-trench was better than that of HEMT without Cu micro-trench. On the other hand, the slopes of curves for HEMT with and without Cu micro-trench are 1.28 and 0.95 mA/mm.K, respectively. It could be resulted that the trench filled high thermal conductive Cu in the substrate causes heat flow from the device to bottom chuck better than with only Si substrate.

For the DC I-V measurement, the voltage applied across drain-source contact V_DS_ varied from 0 to 20 V and the gate bias V_GS_ swept from −8 to 3 V in the step of +3 V. The I_DS_-V_DS_ DC characteristics were shown in Fig. 2(b). It was found that all obtained data about the on-resistance and leakage current of HEMTs in the linear region with trench structure were better than those of HEMTs without trench structure. The inferior performance for the HEMTs without trench was not resulted from the processing. Because the same devices were measured before and after the devices, if there existed leakage current resulted from the processing, it could not be disappeared after the trench fabrication and Cu fill in. Thus, the improved performance for the HEMTs with trench could be resulted from the following reasons: (1) Leakage paths resulted from the threading dislocations from the Si substrate towards epilayers had been interrupted by Si substrate removing, (2) Stress variation by partial Si removing and (3) Junction temperature reducing by the filled Cu. As concerning the above factors, they will be demonstrated by Raman and surface measurements and discussed later.

From the I_DS_-V_DS_ DC characteristics in the saturation region shown in Fig. 2(b), it was found that there existed a negative slope (dI_DS_/dV_DS_), which signified that at a high bias power, carrier mobility degraded for the samples without micro-trench fabrication. This result could be due to the Joule heating effect. The decrease of the I_DS_ saturation current with an increase in ambient temperature and the negative slope from the I-V characteristics were in agreement with the literature^30,31^, which explained the effect of temperature on the performance of high power GaN devices. Correspondently, the drain saturation current at V_GS_ = 4 V increased from 406.92 to 476.42 mA/mm (@ V_DS_ = 10 V), indicating an improvement of 17% for the sample with a micro-trench filled by Cu metal. From the negative conductance (dI_DS_/dV_DS_) slope, it was observed that at a higher gate-source bias of 4 V, the drain current I_DS_ decreased by 18% for the sample with a 150 μm Si substrate. In contrast, the I_DS_ decreased by only 9.5% for the sample with the micro-trench structure. This result demonstrated that the micro-trench structure filled with Cu metal could efficiently remove heat from the HEMT device.

In another method to investigate the self-heating effect, a double-pulsed measurement was done at room temperature. For the pulsed measurement, the narrow pulse width (500 ns) and a very low duty cycle (0.01%) were set respectively at V_DS_ and V_GS_ simultaneously to make the self-heating effect negligible. The drain-source bias V_DS_ (swept from 0 to 15 V) and gate-source bias V_GS_ = 5 V were the same as the DC bias setup. The I_DS_ difference between the DC and the pulsed measurement ∆I_DS_ caused by self-heating effect could be expressed as follows^28,32^:

1 $${\Delta {\rm{I}}}_{{\rm{DS}}}={{\rm{I}}}_{{{\rm{DS}}}_{{\rm{.Pulsed}}}}-{{\rm{I}}}_{{{\rm{DS}}}_{{\rm{.DC}}}}=\frac{{\Delta {\rm{T}}}_{{\rm{channel}}}}{{\rm{\theta }}}$$

where ∆T_channel_ is the raised temperature in the channel due to high drain bias and θ is the linear fit inverse slope of I_DS_ verses T_ambient_ and its unit is mm.K/mA. From the above equation, it was obvious that at high bias power, due to the rise in channel temperature, the I_DS_ difference between the pulse and the DC measurement increased. From the measurement, the drain current I_DS_._DC_ was 21% less with respect to I_DS_._Pulsed_ at V_GS_ = 4 V and V_DS_ = 10 V for the sample with a 150 μm Si substrate. Correspondently, the I_DS.DC_ was only approximately 8% less compared to I_DS_._Pulsed_ for the sample with the Cu filled micro-trench structure, as shown in Fig. 2(c). The smaller ∆I_DS_ for the sample with the micro-trench structure indicated again that the thermal dissipation was better than that of the sample with a 150 μm Si substrate. Moreover, it is worthy to mention that samples with trenches have higher I_D_ in the linear region for measurement in pulse. With pulse of 500 ns and duty cycle of 0.01%, the self-heating in the linear region (Fig. 2(c), dash curves, V_DS_ < 6 V) will be very minimal. It could be resulted from the partial Si substrate being removed and resulted in the stress variation. It could contribute to improving the performance in the linear region of I_D_-V_D_ measurement under pulse and DC for the device with trench structure. As concerning the strain variation, it has been demonstrated by Raman measurement.

Figure 2(d) shows the I_DS_ and transconductance (g_m_) as function of V_GS_ at a constant drain-source bias V_DS_ of 5 V for the samples with the micro-trench structure as compared with the samples with a 150 μm Si substrate. As gm represents the slope (dI_DS_/dV_GS_), a larger I_DS_ variation with V_GS_ bias from −2 to 2 V was observed with the copper-filled trench sample. The peak transconductance (gm) changed from 92 to 116 mS/mm at V_DS_ = 5 V and the threshold voltage V_th_ = −3.8 V remained almost same with fabrication of the micro-trench structure. Inset of Fig. 2(d) show the gm variation among devices. The variation is 101–141 mS/mm and 85–128 mS/mm for devices with and without trench, respectively. Even there existed the variation of gm, the performance of device with micro-trench is always better than that of device without micro-trench. The above results demonstrated an improvement of GaN-on-Si HEMT performance due to effective heat removal.

**Figure 3:**
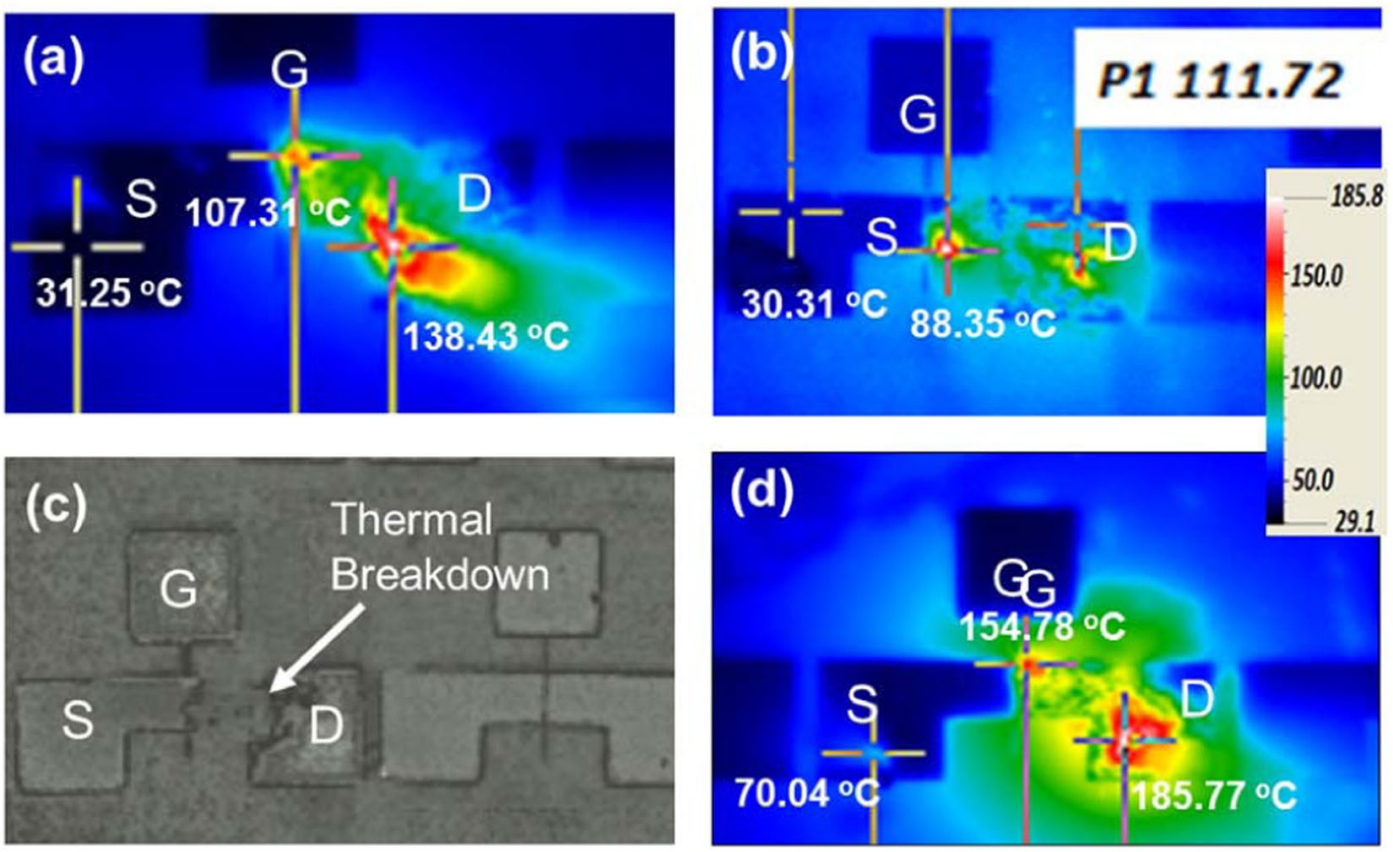
IR Thermography measurement on the sample. (**a,b**) Temperature map on the channel of the device without and with a Cu filled micro-trench at a power density of 11.57 and 12.61 Wmm^−1^. (**c,d**) Thermal breakdown in the HEMT without a trench as compared to no breakdown with a trench at an applied bias of V_DS_ = 200 V.

It was important to measure the surface temperature of the above two samples. Infrared (IR) thermometry was used to investigate high-spatial-resolution temperature profiles over the active area of the GaN-based devices^33,34^. IR images, as shown in Fig. 3, were collected for measurement of the temperature across the active channel of the device by varying the drain-source bias V_DS_ from 0 to 200 V without gate bias. Figure 3(a,b) are thermometric images at the drain and source edge of the channel at a power dissipation of 11.57 and 12.61 W/mm for the sample before and after fabrication of the micro-trench. From the temperature distribution, it was evident that the heat generation was highly localized on the drain side of the channel. Before implementation of the trench, temperatures of about 31.25 and 138.43 °C were observed at the source and drain side of the channel at a power dissipation of 11.57 W/mm. In contrary, with little more high-power dissipation of 12.61 W/mm, the temperatures at the source and drain edge were 30.11 and 111.72 °C in the trench structure. From IR thermometry, the lateral temperature profile of the device was studied. The lateral (in-plane) temperature profiles are 4.53 and 5.95 °C/μm for the HEMT with and without trench structure. Obviously, the temperature profile of the HEMT with Cu-filled trench is lower than that of HEMT without Cu-filled trench. This is because the filled Cu can effectively remove heat. Moreover, it was found that the hotspot is mostly located in drain side of the channel for the device without Cu-filled trench. Correspondently, the heat can be spread and temperature can be reduced for the HEMT with Cu-filled trench as compared with that without Cu-filled trench. This indicated that the improvement in the drain saturation current and transconductance came from the reduction of the self-heating effect. On the other hand, it was found the maximum temperatures seem to occur close to the drain pad and not at the drain-edge of the gate. It is because the measurement of the temperature across the active channel of the device by varying the drain-source bias V_DS_ from 0 to 200 V without gate bias. If the gate was biased, the maximum temperature will be along the gate-edge of the drain side. At a very high drain-source bias of V_DS_ = 200 V thermal breakdown occurred in the normal sample, whereas in the case of the micro-trench device, a temperature of 185 °C was observed, as shown in Fig. 3(c,d). The power dissipation, calculated as P_D_ = V_DS_ × I_DS_, significantly influenced the channel temperature, and at a high power dissipation, T_channel_ was reduced significantly with the micro-trench structure.

**Figure 4:**
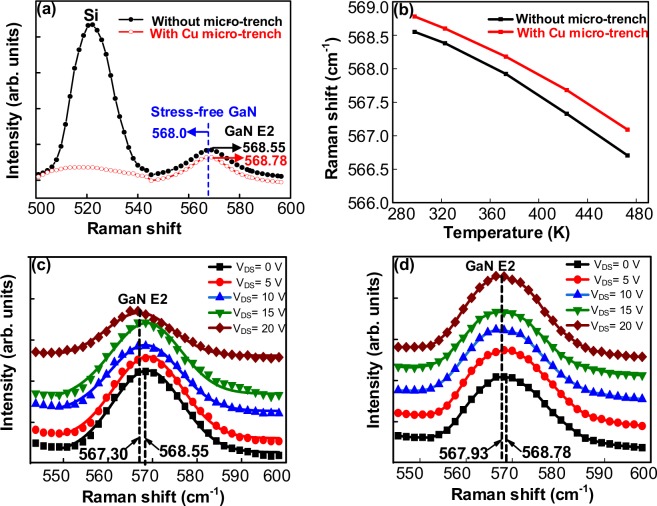
Raman thermometry for validation of temperature rise in the device structure. (**a**) Si and GaN E2 frequency peak (the GaN E2 peak was used for monitoring the channel temperature of the HEMT), (**b**) Temperature dependence of the Raman frequency for the active E2 mode in GaN, (**c**) GaN E2 peak shift in the AlGaN/GaN HEMT without micro-trench fabrication and (**d**) Smaller peak shift in the HEMT with copper filled micro-trench, indicating a reduction in the temperature rise at the same power density.

Micro-Raman thermometry has been another most widely used technique to demonstrate thermal management in GaN power device. Device self-heating effect was investigated by measuring Raman spectra for the devices with and without trench structures. It is worthy to mention that the laser power is below 2 mW for Raman spectroscopy measurement. The laser induced heating is negligible compared to power dissipation (11 Wmm^−1^) by HEMT. The relationship between temperature T and the Raman peak position in GaN can be expressed as^35^:

2 $${\rm{\omega }}({\rm{T}})={{\rm{\omega }}}_{0}-\frac{{\rm{A}}}{\exp ({{\rm{Bhc}}{\rm{\omega }}}_{0}/{\rm{KT}})-1}$$

where. ${{\rm{\omega }}}_{0}$ is the Raman peak at 0 K, A and B are fitting parameters, K is Boltzmann’s constant, h is Plank’s constant, and c is the speed of light. From the above equation, it was expected that the Raman peak would shift to a lower wave number with the rising temperature across the channel region at high power operation and with low thermal dissipation. Figure 4(a) shows the spectra obtained from Raman spectroscopy on the GaN-on-Silicon HEMT structures, in which the peaks of the GaN E2 (high) were selected at 568.55 and 568.78 cm^−1^ to monitor the channel temperature for devices both with and without Cu filled micro-trenches. It is worthy to mention that the shift in the E2 Raman peak could be resulted from the piezoelectric strain which was caused by applied voltage. However, the piezoelectric strain always occurs along the <0001> direction in the GaN epilayers. In this work, the applied voltage was along the width of the device between gate and drain. So the piezoelectric strain can be almost negligible in this measurement. The E2 frequency deviation from the freestanding bulk GaN (568 cm^−1^) sample was due to the compressive stress^36^ effect of the epilayer and trench structure. The measurements were carried out in a back-scattering configuration under a 532 nm laser excitation source at room temperature. This sub-band gap laser source prevented heating of the GaN due to laser light absorption. The laser source, with a beam diameter of 1.3 μm, was focused in between the gate and drain region of the channel, where hot spots were expected to exist. The GaN E2 phonon frequency was extracted using the Lorentz fitting method with a spectral resolution better than 0.1 cm^−1^.

Figure 4(b) shows the Raman shift as function of temperature. This experiment was done to measure Raman peak by keeping the sample inside chamber and vary the temperature in chamber from 280 to 480 K. The temperature was controlled and measured by a thermocouple. By fitting the graph of the Raman phonon frequency and the temperature, as shown in Eq. (2), we extracted ω_0_ = 569.25 cm^−1^, a = 23.0183 cm^−1^, and b = 1.4168 for the device with the trench structure, and ω_0_ = 569.08 cm^−1^, a = 27.48 cm^−1^, and b = 1.4643 for the device without the trench structure. The voltage across drain-source V_DS_ was swept from 0 to 20 V in steps of 5 V and no voltage was applied in between the gate and the source. With the increase of V_DS_, the Raman peak shifted towards a lower wavenumber due to the self-heating effect at high bias power for these two samples. It was found that the peak of GaN E2 shifted from 568.55 to 567.30 cm^−1^ as the V_DS_ operated from 0 V to 20 V for the device without the trench structure. In contrast, the peak of GaN E2 shifted from 568.78 to 567.93 cm^−1^ as the V_DS_ operated from 0 V to 20 V for the device with the Cu filled trench structure. From Eq. (2) and the Raman peak shift shown in Fig. 4(c,d), we estimated at power density of approximately 10.6 W mm^−1^, the temperature rise in channel region was 124 °C and 102 °C for the devices without and with Cu filled micro-trenches, respectively. This result again demonstrated that Si with a Cu filled micro-trench structure could effectively reduce the hotspot temperature in AlGaN/GaN HEMTs. Noted that the Raman signal is a volumetric average, the channel temperature should be higher than that estimated by Raman signal.

## Conclusions

This study presented the results of electrical characterization, infrared thermography imaging, and Raman spectroscopy for GaN-on-Si HEMTs before and after Cu filled micro-trench fabrication. From the IR thermometry, the hot spots were identified at the drain side of the channel as the dominant factor in the self-heating effect. From Micro-Raman spectroscopy, we found the temperature at the hot spot was reduced by 22 °C with the fabrication of Cu-filled trench in the Si substrate with almost the same bias power. From the electrical measurements, the improvement of the drain saturation current and the transconductance were reported as 17% and 26%, and the threshold voltage remained almost constant. The pulse I-V characterization study revealed a difference in I_DS_ between DC, and the pulsed measurement was reduced to 8% from 21% with the implementation of the micro-trench. Hence, the results demonstrated that a Cu filled micro-trench structure is an efficient technique to enhance heat dissipation and improve the power performance of GaN-based semiconductor devices.

## Methods

### Fabrication of HEMT devices

The GaN/AlGaN HEMT device grown by metalorganic chemical vapor deposition was fabricated on epitaxial layers consisting of AlN/GaN buffer/transition layer of thickness 4.5 μm, a 20 nm Al_0.27_Ga_0.73_N barrier layer, and a 2 nm GaN cap layer with 6 inch diameter Si (111) substrate. The Hall mobility and sheet electron concentration measured at room temperature were 1970 cm^2^/Vs and 9.7 × 10^12^ cm^−2^, respectively. Device fabrication began with mesa isolation in a Cl_2_/BCl_3_ inductively coupled plasma (ICP) reactive ion etching system (15/15 sccm Cl_2_/BCl_3_, 150 W source power, 50 W RF power, and 1.5 nm/sec etch rate) for a 160 nm deep mesa. The ohmic contacts for the drain (D) and source (S) electrodes were formed by Ti/Al/Ni/Au metal stacks (30/180/40/100 nm) using an E-beam evaporation system and were subsequently annealed at 850 °C using rapid thermal annealing in an N_2_ environment for 60 seconds. Lastly, the 25/150 nm thick Ni/Au for the Schottky gate contact was formed by E-beam evaporation and the lifting off method. The asymmetric device dimensions were as follows: a source-drain distance (L_SD_) of 18 μm; a drain-gate distance (L_DG_) of 10 μm; and a source-gate distance (L_SG_) of 5 μm. The gate length and width of the HEMT devices under study were 3 μm and 180 μm, respectively.

### Micro-trench implementation after HEMT fabrication

After the device processing, Si substrate was reduced to 150 μm for effective implementation of the micro-trench structure. First, a carrier glass plate was bonded to the device side of the silicon substrate using wax as an adhesive layer. The chemical-mechanical planarization process was applied for lapping and polishing of the Si substrate using alumina slurry. Initially, 9 μm size alumina powder (and thereafter 3 μm) was used to obtain 150-um-thick Si substrate. Further, a 0.3 μm-sized slurry powder was used in the polishing process to optimize the surface roughness. After thinning and polishing, the glass plate was debonded and an ultrasonic bath was carried out with acetone, Isopropyl Alcohol and DI water to clean sample. Devices were fabricated on broken wafer of size 2 cm ×2 cm taken from 6 inch epiwafer.

**Figure 5:**
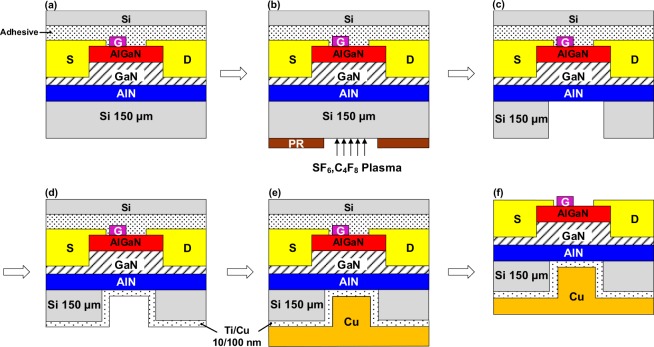
Schematic views of the process flow for micro-trench fabrication in AlGaN/GaN HEMT. (**a**) Temporary bonding of Si wafers on the front side of the device using adhesive, (**b**) Lithography to expose micro-trench pattern for Si etching using SF_6_/C_4_F_8_ Plasma, (**c**) Removal of photoresist and cleaning after 150 µm Si deep etching, (**d**) Deposition of the Ti/Cu seed layer by sputtering, (**e**) Filling the micro-trench with Cu by electroplating and (**f)** Removal of the temporary Si substrate.

The process flow to fabricate copper filled micro-trench structure in the backside of GaN/AlGaN HEMT is illustrated in Fig. 5(a–f) sequentially. After the transistor implemented on the front side of GaN-on-Si epiwafer, the micro-trench structure was fabricated in the backside of wafer on the silicon substrate. First, a temporary Si wafer was bonded to the front side of the sample as shown in Fig. 5(a) to protect the device. Next, a photolithography pattern and deep reactive ion etching (DRIE) through the Bosch process were performed to fabricate a deep rectangular micro-trench of length 200 μm, width 180 μm and depth of 150 μm as shown in Fig. 5(b). The etching chemistry used were SF_6_ and C_4_F_8_ plasmas in the anisotropic etch process with 2000 W of ICP power. The gas flow rate of SF_6_ and C_4_F_8_ were maintained at 800 and 10 sccm throughout the process to prevent sidewall etching and achieve etch rate of 5 μm/min. The trench structure was patterned below the hot-spot region that located in channel and drain region of the device. Once the deep etching is finished, photoresist was removed and sample was rinsed gently in solvent and DI water to remove chemical residues. A Ti/Cu seed layer with a thickness of 10/100 nm was deposited using a sputtering system at high pressure for better adhesion prior to the electrodeposition of the Cu, as shown in Fig. 5(d). The electrodeposition experiments at different current densities were studied and optimized to achieve defect-free filling. Finally, cost-effective low-temperature electroplating was performed to fill the micro-trench by setting the forward current density at 10 mA/cm^2^ to result in a 150 µm Cu thickness, as shown in Fig. 5(e).

### Electrical and thermal characterization setup

To evaluate the etched trench, cross-sections of the samples were examined using SEM. The electrical properties were measured using an Agilent B1505A parameter analyzer at room temperature. Advanced Thermo TVS-500EX IR thermometry was used to investigate high-spatial-resolution temperature profiles over the active area of the GaN-based devices. In this setup, the surface temperatures of the transistor could be measured by detecting infrared radiation leaving the surface through focusing the microscope objective onto the detector. The temperature distribution of the entire field of view at the same time could be examined with an IR microscope. Micro-Raman spectroscopy was performed using a Renishaw micro-Raman system to compare the rise in channel temperature between devices with and without a Cu filled micro-trench structure by measuring the temperature-dependent shift in the Raman peak position.
